# A high-fat diet disrupts the hepatic and adipose circadian rhythms and modulates the diurnal rhythm of gut microbiota-derived short-chain fatty acids in gestational mice

**DOI:** 10.3389/fnut.2022.925390

**Published:** 2022-09-28

**Authors:** Lu Ding, Jieying Liu, Liyuan Zhou, Xinmiao Jia, Shunhua Li, Qian Zhang, Miao Yu, Xinhua Xiao

**Affiliations:** ^1^Department of Endocrinology, Key Laboratory of Endocrinology, Ministry of Health, Peking Union Medical College Hospital, Chinese Academy of Medical Sciences and Peking Union Medical College, Beijing, China; ^2^Department of Medical Research Center, Peking Union Medical College Hospital, Chinese Academy of Medical Sciences and Peking Union Medical College, Beijing, China

**Keywords:** gut microbiota, circadian rhythm, SCFAs, high-fat diet, glucose and lipid metabolism, gestational obesity

## Abstract

The prevalence of gestational obesity has reached epidemic proportions. Evidence supported that the interactions between the gut microbiota and circadian clocks far reached, affecting host metabolism. Our study aimed to investigate the effect of a high-fat diet (HF) on the hepatic and adipose circadian rhythms in gestational mice and to explore the role of gut microbiota-derived short-chain fatty acids (SCFAs) in mediating the effects. C57BL/6 female mice were randomly fed a standard chow diet (Ctr) or HF prior to and during pregnancy. Samples were collected every 4 h over 24 h (six time points), and 16S rRNA and metabonomics were carried out. Rhythmic patterns were identified and compared using CircaCompare. The results showed that the HF before and during pregnancy significantly induced obesity and worsen glucose tolerance, insulin sensitivity, and lipid metabolism in the gestational mice. Furthermore, the HF significantly disrupted the rhythmic pattern of hepatic and adipose circadian clock genes and downstream metabolic genes. Importantly, our results revealed that the HF altered the diurnal rhythm of the gut microbiota in a diverse manner, which was assessed across three categories: phase shift, loss rhythmicity, and gained rhythmicity. We report here, for the first time, a parallel alteration of the rhythmic phase of butyric acid and butyrate-producing *Clostridiaceae_1*, which was confirmed by a positive correlation between them. Overall, our research emphasized the importance of the rhythmicity of gut microbiota-derived SCFAs in mediating circadian disruption in response to the HF in gestational mice, which may provide novel insights into the prevention and treatment of gestational obesity.

## Introduction

The prevalence of gestational obesity has reached epidemic proportions (1). Epidemiologic and animal studies have demonstrated that gestational obesity increased not only the risk of developing gestational diabetes mellitus (2), lipid disorders (3), congenital heart disease (4), and preterm delivery (5, 6) but also the risk of progressing chronic metabolic diseases in the later life of offspring (7–9). With its increasing prevalence and subsequent health outcomes, it is of great importance to uncover the critical mechanisms of gestational obesity.

In mammals, the circadian clock system synchronizes daily rhythms of homeostasis, enabling organisms to temporally coordinate physiology and to align it with geophysical time (10, 11). The ubiquitous rhythmic transcriptional program is controlled by a network of core clock transcription factors, including *circadian locomotor output cycles kaput* (*CLOCK*), *brain and muscle Arnt-like protein* (*Bmal1*), *period* (*Per*), and *cryptochrome* (*Cry*), with *nuclear receptor subfamily 1*, *group D*, and *member 1* (*Rev-erb*α) family stabilizing the core oscillator (12). While the hypothalamic central clock is controlled by light, food consumption is widely considered a major stimulus for peripheral clocks in different organs (13). A high-fat diet (HF) was found to reprogram hepatic circadian clocks *via* the rewiring of specific transcriptional pathways (14). Moreover, an HF induced obesity and inhibited the expression of clock genes in adipose tissues (14). However, tissue responses to an HF are still controversial due to different nutritional compositions, intervention times, and animal models (15–18). Intriguingly, a recent study reported a poor association between the response of local clocks and the tissue rhythmic transcriptome (19). Consequently, the specific underlying mechanism responsible for the disruption of peripheral circadian rhythm remains unclear.

As a non-canonical oscillator, the gut microbiota mainly entrains dietary cues to regulate host metabolism (20). Frazier et al. newly reported that the host C-type lectin antimicrobial peptide *Reg3g* worked with key ileal microbes to induce metabolic disruption in the HF-treated mice model (21). Moreover, accumulating evidence supported that the interactions between the gut microbiota and peripheral circadian rhythm far reached, affecting host metabolism (18, 22). A recent study showed that the diurnal alteration of the gut microbiota in response to an HF reprogrammed hepatic circadian clocks and contributed to obesity in mice compared with mice fed a normal chow diet or a low-fat diet (17). Although some of the hepatic core clock genes (*Bmal1* and *Per2*) remained comparable between groups, the rhythmic transcription of PPAR signaling was altered in the HF-fed mice (17). Importantly, these effects were replicated in germ-free mice that received fecal transplantation of the gut microbiota derived from HF-fed mice, which further confirmed that the gut microbiota modulated hepatic lipid metabolism in response to the HF mainly by activating the host’s circadian rhythmicity of PPAR signaling (14). Research on how the gut microbiota modulates host metabolism by interacting with host circadian clocks has focused on microbially produced metabolites, particularly including short-chain fatty acids (SCFAs). Studies have continued to show that the circadian rhythm of gut microbiota-derived SCFAs has vital effects on the host’s circadian control of metabolism (14, 23). However, research on the mechanism underlying the interaction of gut microbiota and peripheral clocks and how they affect the glucose and lipid metabolism in gestational mice is limited. A clear understanding of the mechanistic changes underlying these processes could have a profoundly beneficial impact on the prevention and treatment of gestational obesity.

Therefore, in the current study, we established a gestational obesity model by feeding mice an HF prior to and during pregnancy. We aimed to investigate the effects of the HF on hepatic and adipose circadian rhythms, emphasizing alterations of the rhythmic pattern of metabolic parameters. In addition, we explored the role of gut microbiota-derived SCFAs in mediating circadian disruption in response to HF in gestational mice.

## Materials and methods

### Animals and experimental scheme

**FIGURE 1:**
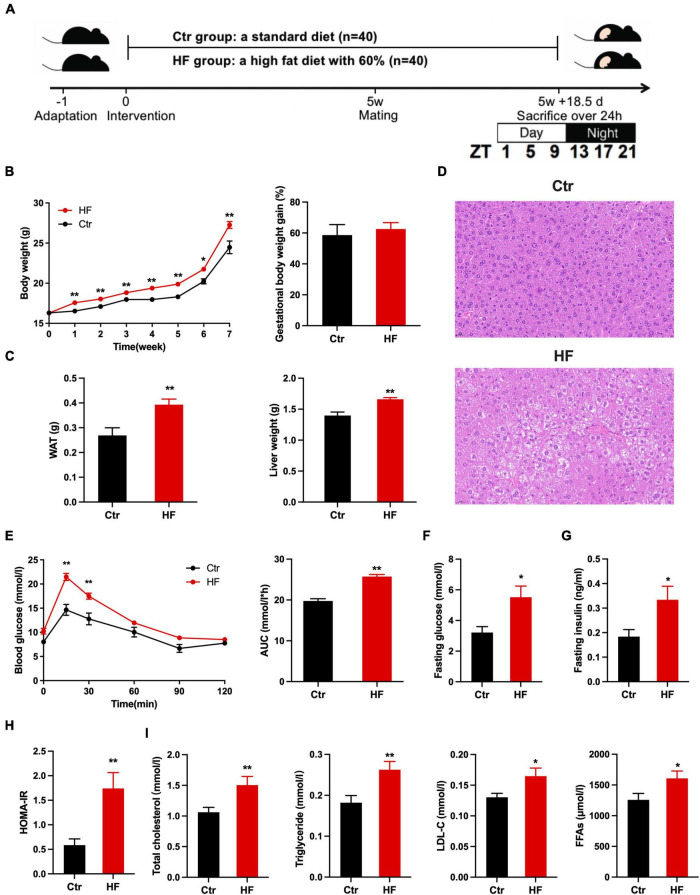
High fat diet induced obesity and metabolic disorders in gestational mice. Mice were fed with a standard chow diet, or a high-fat diet prior to and throughout pregnancy. The morning of vaginal plug confirmed the pregnancy and was considered E0.5. The pregnant mice continued to feed on their respective diets until E18.5. **(A)** Experimental design; **(B)** body weight alteration during the high-fat diet treatment (left) and gestational weight gain (right); **(C)** weight of the WAT (left) and liver (right); **(D)** histological examination of liver tissues; **(E)** oral glucose tolerance test after fasting for 6 h (left) and the area under the curve (right); **(F)** fasting glucose level; **(G)** fasting insulin level; **(H)** HOMA-IR; **(I)** level of TC, TG, LDL-C, and FFAs. Ctr, standard chow diet-fed pregnant mice; HF, high-fat diet-fed pregnant mice; HOMA-IR, homeostasis model assessment of insulin resistance; TC, total cholesterol; TG, triglyceride; LDL-C, low-density lipoprotein cholesterol; FFAs, free fatty acids. Data are expressed as means ± S.E.M. (*n* = 8–12/group). Student’s *t*-test and two-way ANOVA; *p < 0.05 and **p < 0.01.

A total of 80 4-week-old C57BL/6 female mice were purchased from Beijing Huafukang Biotechnology Co., Ltd., (Beijing, China, SYXC-2014-0029). The mice were maintained in a specific pathogen-free (SPF) environment at a controlled temperature of 22 ± 2°C and subjected to a 12:12 light/dark cycle (lights on at 06:00 am, ZT0 = 06:00). After acclimation for 1 week, 5-week-old mice with similar body weights were randomly assigned to one of two groups: a control group (Ctr, *n* = 40), which was fed a standard chow diet (AIN-93G) (15.8% of the calories as fat), and a high-fat diet group (HF, *n* = 40), which was fed a high-fat diet (D12492) (60% of the calories as fat). After 5 weeks of intervention, the C57BL/6 female mice were mated with normal 9-week-old C57BL/6 male control mice. Pregnancy was timed based on vaginal plug formation. The morning of vaginal plug was considered E0.5. Weekly body weight and food consumption were recorded. The pregnant mice continued on their respective diets throughout the pregnancy and were killed every 4 h over 24 h at six different time points of 07:00 (ZT1), 11:00 (ZT5), 15:00 (ZT9), 19:00 (ZT13), 23:00 (ZT17), or 03:00 (ZT21) on E18.5 (Figure 1A). All procedures were performed in accordance with the protocol approved by the Animal Care and Ethics Committee at Peking Union Medical College Hospital (Beijing, China, XHDW-2019-012). All animal operations were conducted in compliance with the National Institutes of Health Guide for the Care and Use of Laboratory Animals.

### Hematoxylin and eosin staining

In brief, liver tissues were fixed in 4% PFA and embedded in paraffin. Then, 4-μm-thick sections of the tissues were prepared and stained with hematoxylin and eosin (HE) for morphological analysis.

### Glucose tolerance tests

For the glucose tolerance test (GTT), the mice fasted for 6 h were orally administered a glucose load (2 g/kg of body weight). Blood glucose levels were monitored by the tail bleeding time before intervention (0 min) and 15, 30, 60, 90, and 120 min after intervention using a glucometer (Bayer, Leverkusen, Germany). The area under the curve (AUC) was calculated, as previously described (24).

### Serum biochemical analyses

For serum isolation, blood samples were collected from the infraorbital retrobulbar plexus of the mice, separated by centrifugation at 3,000 × *g* for 10 min at 4°C, and then stored at −80°C. Total triglyceride (TG), total cholesterol (TC), low-density lipoprotein cholesterol (LDL-C), and free fatty acids (FFAs) were measured, as previously described (25).

### Enzyme-linked immunosorbent assay

Serum insulin was measured using an enzyme-linked immunosorbent assay (ELISA) kit (80-INSMSU-E01, Salem, NH, United States) following the manufacturer’s protocol. The homeostasis model assessment–insulin resistance (HOMA-IR) index was calculated according to the following formula: [fasting insulin concentration (mU/L) × fasting glucose concentration (mg/dL)]/22.5.

### Reverse transcription quantitative PCR (RT-qPCR) experiment

An E.Z.N.A. Total RNA Kit (Life Technologies In., Carlsbad, CA, United States) was used to extract total RNA from liver tissues. cDNA synthesis was performed with 1 μg total RNA using the PrimeScriptTM RT Reagent Kit with gDNA Eraser (RR047A, TaKaRa Bio Inc., Otsu, Shiga, Japan). RT-qPCR was performed using SYBR^®^ Green Master Mix (RR820A, Takara Bio Inc., Otsu, Shiga, Japan) in an ABI PRISM^®^ 7500 Sequence Detection System (Applied Biosystems Inc.). The relative expression levels of mRNAs were calculated using the 2^–ΔΔCt^ method. The first normalization was to the geometrical mean of reference genes. The second normalization was to the mean expression of each condition. Log2 transformation was applied to the mRNA expression for presentation purposes (19). The primer sequences used for RT-qPCR are detailed in Supplementary Table 1.

### Fecal microbiota analysis

Microbial DNA was extracted from the cecal content using a cecal DNA isolation kit (MN NucleoSpin 96 Soi, Germany) following the manufacturer’s protocol. The V3-V4 regions of bacterial 16S rRNA genes were amplified using the primers 338F, 5′-ACTCCTACGGGAGGCAGCA-3′ and 806R, 5′-GGACTACVVGGGTATCTAATC-3′ with the barcode. PCR products were purified using the Qiagen Gel Extraction Kit (Qiagen, Germany) and quantified using a Qubit@ 2.0 Fluorometer (Thermo Scientific, United States). Tags were sequenced on the Illumina NovaSeq platform. Raw tags were quality-filtered under specific filtering conditions to obtain high-quality clean tags (26) according to the QIIME (version 1.9.1^1^) quality control process (27). Operational taxonomic units (OTUs) were clustered with a 97% similarity cutoff using UPARSE (version 7.0.1001^2^) (28). The Silva Database^3^ was used to annotate representative sequences based on the Mothur algorithm (29).

### Short-chain fatty acid measurement

Liver tissues were immediately collected and frozen in liquid nitrogen before GC–MS analysis. The concentration of SCFA metabolites was measured at Shanghai Biotree Biotech Co., Ltd., (Shanghai, China), as described in previous studies (30, 31). In brief, SCFAs, including acetic acid, propionic acid, butyric acid, valerate acid, isobutyric acid, isovaleric acid, and hexanoic acid, were extracted from liver tissues using solvent mixtures containing methyl tert-butyl ether and 2-methylvaleric acid. The aliquot (1 μL) was analyzed by an HP-FFAP capillary column (Agilent, Folsom, CA, United States). With helium as the carrier gas, the initial temperature was set to 80°C for 1 min, subsequently increased to 200°C for 5 min, and then maintained at 240°C for 1 min. The injection, transfer line, quad, and ion source temperatures were 240°C, 240°C, 150°C, and 200°C, respectively, and the electron impact energy was −70 eV. Mass spectrometry data were acquired in the Scan/SIM mode, with an m/z range of 33–150 after a solvent delay of 3.5 min.

### Statistical analyses

Statistics were analyzed by Student’s *t*-test or two-way ANOVA with Tukey’s *post hoc* analyses. A *P*-value < 0.05 was considered statistically significant. Rhythmic features were defined as any feature with periods of 24 h by a *P*-value < 0.05. Specifically, loss in rhythmicity indicated a parameter which was significantly oscillated in the Ctr group, but not significantly oscillated in the HF group (Ctr *p* < 0.05, HF *p* > 0.05). Contrarily, gain rhythmicity indicated a parameter which was not significantly oscillated in the Ctr group but significantly oscillated in the HF group (Ctr *p* > 0.05, HF *p* < 0.05) (32). Phase, MESOR, and amplitude were monitored and compared using the non-linear cosinor regression tool CircaCompare when the parameters significantly oscillated in the two groups (33). Correlation analyses were performed by Spearman and Pearson correlation coefficient tests. All statistical analyses and data visualization were performed with either R v.4.1.1 or GraphPad Prism version 8.0 (GraphPad Software Inc., San Diego, CA, United States). In all bar and line plots, data were presented as the mean ± standard error of mean (S.E.M.).

## Results

### High-fat diet induced obesity in gestational mice

The experimental design of the study is illustrated in Figure 1A. To determine the effects of the HF on gestational mice, female mice were fed with an HF prior to pregnancy for 5 weeks and throughout pregnancy. The results showed that the HF significantly induced body weight gain prior to and throughout pregnancy (*P* < 0.05), although gestational body weight gain was not obviously different between the two groups (Figure 1B). The subcutaneous white adipose tissue (WAT) was significantly increased in the HF group compared with the Ctr group (*P* < 0.01, Figure 1C). Simultaneously, the liver weight was dramatically increased in the gestational mice of the HF group compared with that of the Ctr group (*P* < 0.01, Figure 1C), which was consistent with the increase in hepatocyte size (Figure 1D). These results revealed that the HF increased body weight gain, liver weight, and subcutaneous WAT weight, leading to obesity in the gestational mice.

### The effects of high-fat diet on glucose and lipid metabolism in gestational mice

Since the HF significantly programmed the body weight and body composition in the gestational mice, we further detected the effects of the HF on glucose and lipid metabolism in the gestational obese mice. In mice on the HF, the oral GTT significantly elevated blood glucose at 15 min and 30 min after the administration of glucose (*P* < 0.01), and the calculated AUC of the OGTT result was also dramatically elevated in the HF group compared with the Ctr group (*P* < 0.01, Figure 1E). In addition, the HF led to significantly increased fasting glucose and insulin levels in the gestational mice (*P* < 0.05, Figures 1F,G), which resulted in a remarkable elevation in the HOMA-IR index (*P* < 0.01, Figure 1H). Concomitantly, TC, TG, LDL-C, and FFA levels were significantly elevated in the HF group (*P* < 0.05, Figure 1I). These results demonstrated that the HF markedly impaired glucose and lipid homeostasis and insulin sensitivity in the gestational obese mice.

### High-fat diet disrupted the hepatic and adipose circadian rhythms in gestational mice

**FIGURE 2:**
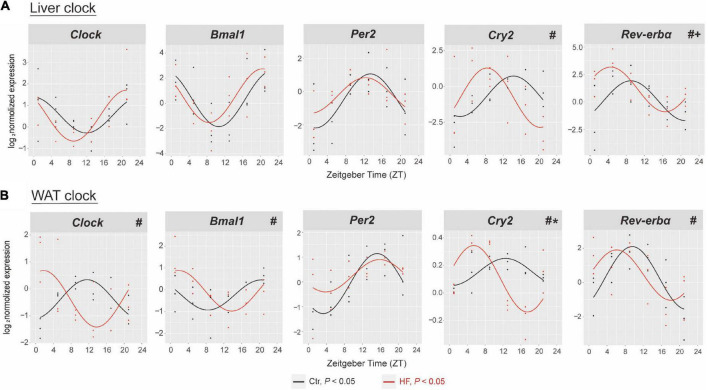
High-fat diet disrupted the hepatic and adipose circadian rhythm in gestational mice. Mice were fed with a standard chow diet or a high-fat diet prior to pregnancy for 5 weeks, and throughout pregnancy. The morning of vaginal plug confirmed the pregnancy and was considered E0.5. On E18.5, the mice were killed at 4-h intervals over 24 h. Rhythmic pattern of circadian clock genes in the **(A)** liver and **(B)** WAT. The dots mark individual measurements in each zeitgeber time (ZT), and line represents cosinor regression fit. Rhythmic pattern of genes with significant differences in phase (#), amplitude (*), or MESOR (+) derived from CircaCompare (*n* = 2–3/time point). Ctr, standard chow diet-fed pregnant mice; HF, high-fat diet-fed pregnant mice; WAT, white adipose tissue.

To address the role of the circadian rhythm in response to the HF, we first characterized the oscillation of the circadian clock genes of metabolic peripheral tissues liver and WAT and compared their rhythmic pattern in the gestational mice. The expression of *Clock*, *Bmal1*, *Per2*, *Cry2*, and *Rev-erv*α exhibited periodic oscillations after both feeding intervention in the liver and WAT (all *P* < 0.05, Figure 2). As shown in Figure 2A, the rhythmic pattern of hepatic *Cry2* and *Rev-erb*α was significantly phase-advanced in the HF group compared with the Ctr group (both *P* < 0.05). Consistently, the *Clock*, *Bmal1*, *Cry2*, and *Rev-erb*α phases were significantly advanced in the WAT (all *P* < 0.05, Figure 2B). However, the rhythmic pattern of clock genes also displayed tissue specificity: the MESOR of hepatic *Rev-erb*α revealed significant elevation after HF treatment compared with the Ctr group (*P* < 0.05, Figure 2A), while the amplitude of *Cry2* was strikingly higher in the HF group than in the Ctr group in the WAT (*P* < 0.05, Figure 2B). There was no dramatic difference between the feeding protocols for either phase, MESOR or amplitude for the rhythmic pattern of hepatic *Clock*, *Bmal1*, and *Per2* and adipose *Per2*.

**FIGURE 3:**
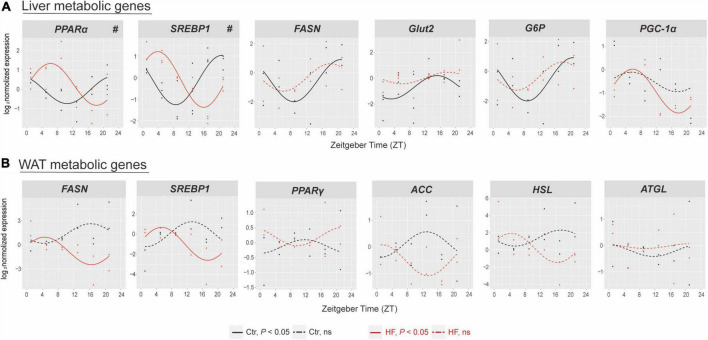
The effects of high-fat diet on metabolic genes of liver and white adipose tissue in gestational mice. Mice were fed with a standard chow diet or a high-fat diet prior to pregnancy for 5 weeks, and throughout pregnancy. The morning of vaginal plug confirmed the pregnancy and was considered E0.5. On E18.5, the mice were killed at 4-h intervals over 24 h. Rhythmic pattern of metabolic genes in the **(A)** liver and **(B)** WAT. Significant rhythms (*P* < 0.05) are denoted by the continuous line, and non-significant rhythms are denoted by the dotted line. The dots mark individual measurements in each zeitgeber time (ZT), and line represents cosinor regression fit. Rhythmic pattern of genes with significant differences in phase (#) derived from CircaCompare (*n* = 2–3/time point). Ctr, standard chow diet-fed pregnant mice; HF, high-fat diet-fed pregnant mice; WAT, white adipose tissue.

We next determined the expression of selected genes involved in glucose and lipid metabolic physiology in the liver and WAT. The results showed that the rhythmic patterns vary among different metabolic genes in different tissues. In the liver (Figure 3A), the expression of *PPAR*α and *SREBP1* exhibited rhythmic patterns in both feeding protocols, and both were significantly phase-advanced in the HF group compared with the Ctr group (all *P* < 0.05). By contrast, the expression of *FASN*, *Glut2*, and *G6P* lost diurnal rhythmicity after HF treatment, while the expression of *PGC-1*α merely oscillated in the HF group. In the WAT (Figure 3B), the expression of *SREBP1* and *FASN* merely oscillated in the HF group, and the expression of *PPAR*γ, *ACC*, *HSL*, and *ATGL* did not exhibit statistical diurnal rhythmicity on either feeding intervention. These observations suggested that disruption of the peripheral circadian rhythm had a significant impact on metabolic physiology.

### The diurnal rhythm of gut microbiota was altered in a diverse manner in high-fat-treated gestational mice

**FIGURE 4:**
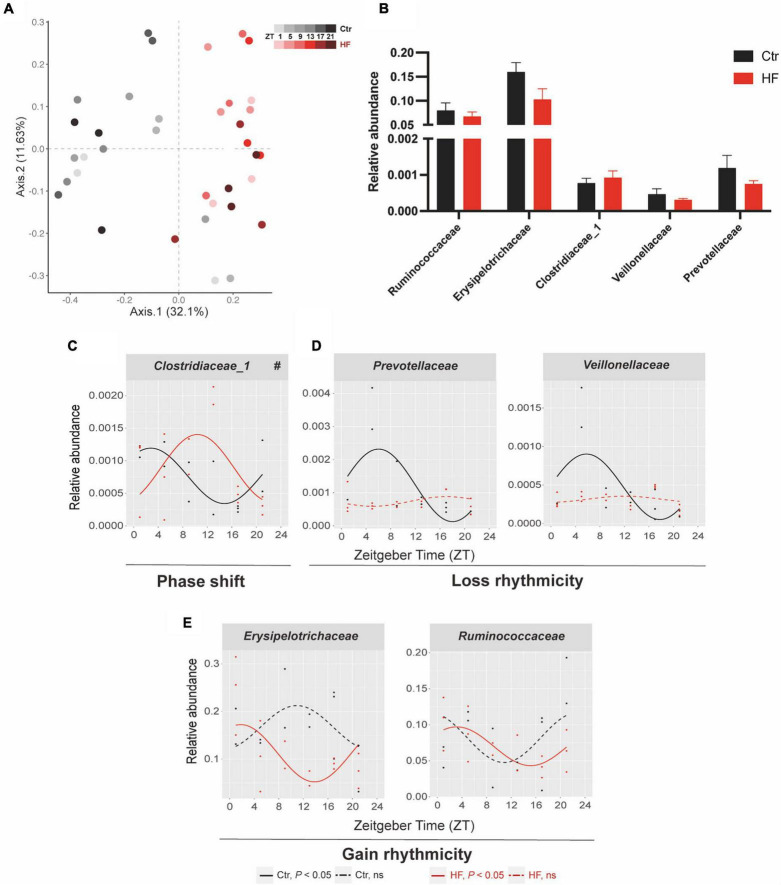
Diurnal rhythm of gut microbiota was altered in a diverse manner in HF-treated gestational mice. Mice were fed with a standard chow diet, or a high-fat diet prior to pregnancy for 5 weeks, and throughout pregnancy. The morning of vaginal plug confirmed the pregnancy and was considered E0.5. On E18.5, the pregnant mice were killed at 4-h intervals over 24 h. **(A)** Bray–Curtis PCoA. Shading represents different time points as indicated. **(B)** The relative abundance and **(C–E)** the diurnal patterns of microbiota. Significant rhythms (*P* < 0.05) are denoted by the continuous line, and non-significant rhythms are denoted by the dotted line. The dots mark individual measurements in each zeitgeber time (ZT), and line represents cosinor regression fit. Rhythmic pattern of genes with significant differences in phase (#) derived from CircaCompare (*n* = 2–3/time point). Data of bar chart are expressed as means ± S.E.M. (*n* = 12/group) using Student’s *t*-test. Ctr, standard chow diet-fed pregnant mice; HF, high-fat diet-fed pregnant mice.

The gut microbiota has been identified as a crucial participant involved in host circadian rhythms in response to dietary cues and itself also undergoes diurnal oscillation (20). We next performed 16S rRNA gene sequencing to examine whether the gut microbiota plays a role in regulating the peripheral circadian rhythm in the gestational mice. PCoA showed that the HF markedly altered the microbial community composition across different time points compared with the Ctr group (Figure 4A). The rhythmic patterns of the relative abundance varied among the different microbial families, although there was no significant difference in family-level relative abundance between the two groups (Figure 4B). One of the families, *Clostridiaceae_1*, exhibited a diurnal rhythm on both feeding protocols, and its rhythmic pattern was dramatically phase-delayed in the HF group compared with that in the Ctr group (*P* < 0.05, Figure 4C). Simultaneously, *Veillonellaceae* and *Prevotellaceae* lost rhythmicity in the HF treatment group (Figure 4D), while *Erysipelotrichaceae* and *Ruminococcaceae* gained rhythmicity in the HF group (Figure 4E). Collectively, these data revealed that the HF altered the diurnal rhythm of gut microbiota in a complex and diverse manner in the gestational mice.

### High-fat diet modulated the diurnal rhythm of gut microbiota-derived short-chain fatty acids

**FIGURE 5:**
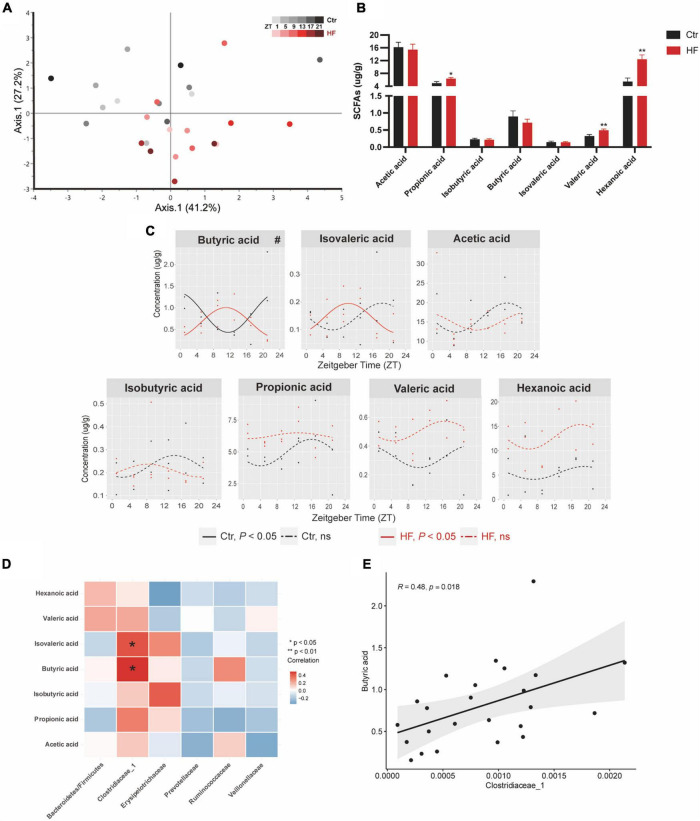
High-fat diet modulated the diurnal rhythm of gut microbiota-derived short-chain fatty acids. Mice were fed with a standard chow diet, or a high-fat diet prior to pregnancy for 5 weeks, and throughout pregnancy. The morning of vaginal plug confirmed the pregnancy and was considered E0.5. On E18.5, pregnant mice were killed at 4-h intervals over 24 h. **(A)** Euclidean distance PCoA. Shading represents different time points as indicated. **(B)** Relative abundance and **(C)** diurnal patterns of SCFAs. **(D)** Heatmap of correlation analysis between SCFAs and the altered family. **(E)** Correlation analysis between butyric acid and *Clostridiaceae_1*. Significant rhythms (*P* < 0.05) are denoted by the continuous line, and non-significant rhythms are denoted by the dotted line. The dots mark individual measurements in each zeitgeber time (ZT), and line represents cosinor regression fit. Rhythmic pattern of genes with significant differences in phase (#) derived from CircaCompare (*n* = 2–3/time point). Data of bar chart are expressed as means ± S.E.M. (*n* = 12/group) using Student’s *t*-test; *p < 0.05 and **p < 0.01. Ctr, standard chow diet-fed pregnant mice; HF, high-fat diet-fed pregnant mice; SCFAs, short-chain fatty acids.

Microbiota-derived SCFAs are regulatory controllers of host metabolism, and they are transported by the portal vein (34). Since SCFAs are mainly absorbed by the liver, we monitored the hepatic SCFA concentration by GC-MS. As indicated in Figure 5A, PCoA demonstrated that the HF altered the concentration of SCFAs across different time points compared with that of the Ctr group (Figure 5A). The hepatic concentration of propionic acid, valeric acid, and hexanoic acid was significantly increased in the HF group compared with the Ctr group (all *P* < 0.05), while acetic acid, isobutyric acid, butyric acid, and isovaleric acid showed no difference between the groups (Figure 5B). Among all the SCFAs (Figure 5C), butyric acid exhibited a diurnal rhythm in both feeding protocols and was significantly phase-delayed in the HF group compared with the Ctr group (*P* < 0.05). In addition, isovaleric acid increased rhythmically after HF treatment. Other SCFAs did not exhibit a statistically significant diurnal rhythm in either feeding intervention. The association between SCFAs and the gut microbiota was confirmed by correlation analysis (Figures 5D,E). The results showed that butyric acid and isovaleric acid were significantly positively correlated with *Clostridiaceae_1*.

## Discussion

An adverse gestational metabolic status exposes the fetus to a suboptimal intrauterine environment, which can disrupt fetal development and influence long-term impacts on health and disease in offspring (35, 36). Such a causal relationship is encompassed in the concept of the Developmental Origins of Health and Disease (DOHaD) (37). Our previous studies suggested that a maternal HF before and during pregnancy and lactation contributed to glucose and lipid metabolic disorders in dams and offspring (24, 38). Here, we further confirmed that the HF before and during pregnancy significantly induced obesity and dramatically worsen glucose tolerance, insulin sensitivity, and lipid metabolism in the gestational mice.

The liver and adipose tissues are core sites for the central control of glucose and lipid metabolism (13). Studies have suggested that among different peripheral tissues, daytime feeding strongly inverted the local clocks in the liver and WAT compared with freely fed animals (19), indicating that the circadian clock in the liver and adipose tissue are most susceptible to different nutritional regimes. To date, the effects of an HF on the circadian clock in the liver and WAT are still controversial due to complexity imposed by different nutritional compositions, intervention times, and animal models (15–18). In this study, we fed the C57BL/6 female mice a 60% (kcal) fat diet prior to and during pregnancy. Our results showed that the HF significantly advanced the rhythmic phase of hepatic *Cry2* and *Rev-erb*α and adipose *Clock*, *Bmal1*, *Cry2*, and *Rev-erb*α in the gestational mice. As one of the core clock machineries, *Cry* regulated hepatic gluconeogenesis through activating the G protein-coupled receptor, which blocked the glucose-mediated increases in the intracellular adenosine 3′,5′-cyclic monophosphate (cAMP) concentrations and in the PKA-mediated phosphorylation of Creb (39). The nuclear receptor *Rev-erb*α has been shown to unite with core circadian clock genes and other components of the principal feedback loop, contributing to the clock function in driving rhythmic expression patterns (40). Cistromic analysis has shown that dual depletion of *Rev-erb*α/β in mice profoundly disrupts lipid metabolic gene networks, emphasizing its vital role in maintaining lipid homeostasis (12). Moreover, diverse nutrients may activate different metabolic and physiological signals, all of which are potential zeitgebers (41, 42). The responses and interactions between different signals can vary for different organs. Therefore, this partly explains the varied extent of the phase shift of the circadian clock in the liver and WAT. To the best of our knowledge, this is the first study to report a phase shift in the hepatic and adipose circadian clock in HF-treated gestational mice. These outcomes may extend the understanding of how peripheral circadian clocks respond to the HF in the gestational mice.

Oscillations of the core circadian clock must be transmitted *via* transcriptional signals among multiple clock-controlled genes to drive downstream rhythmic outputs (11). However, a recent study reported a poor association between the response of the clock and the tissue rhythmic transcriptome (19). Consistent with this study, our findings revealed that the alteration of metabolic genes did not perfectly match the change in the tissue clock on the HF. In the liver, the nuclear receptor *PPAR*α, a crucial factor in regulating dynamic lipid metabolism (43), and the lipogenesis gene *SREBP1* were phase-advanced in the HF group. Furthermore, the fatty acid synthase *FASN*, glucose transporter *Glut2*, and the glycolysis key enzyme *G6P* lost rhythmicity statistically, while the gluconeogenesis gene *PGC-1*α gained rhythmicity in the liver of the HF group compared with that of the Ctr group. In the WAT, *FASN* and *SREBP1* gained rhythmicity in the HF group compared with the Ctr group. Neither the master regulator of adipogenesis, *PPAR*γ, nor the lipogenesis gene *ACC*, and the lipidolysis genes *HSL* and *ATGL* exhibited rhythmicity statistically in both groups. Taken together, we found that the HF resulted in a significant phase shift in hepatic and adipose circadian clock genes and downstream metabolic genes, which is different from the non-pregnant HF-fed mice (44, 45). Studies have confirmed that progesterone and estrogen exhibited rhythmicity (46, 47). Simultaneously, evidence has showed that progesterone altered genes along circadian rhythm signaling and hypothalamic–pituitary–gonadal axes (48). Since the gestational mice experience dramatic change in the level of sex hormone, these may partially explain the difference between the circadian manner in the pregnant mice and non-pregnant mice, although the circadian rhythm of the sex hormone need to be further determined.

The gut microbiota is confirmed to exhibit diurnal rhythmicity in relative abundance and functional level, which has a major influence on the circadian clock network and host metabolism (17, 49). Unlike other peripheral organs, microbial oscillators neither entrain on photic cues nor follow the control of the core clock machinery. Dietary cues are the primary driver in modulating the rhythmic pattern of the gut microbiota (20). Since the rhythmic patterns of metabolic genes were not entirely regulated by their core clock, we hypothesized that the gut microbiota played a role in modulating the host peripheral circadian clock and downstream genes. Intriguingly, we found that the HF altered the diurnal rhythm of gut microbiota in a diverse manner, which we classified into three categories: (1) phase shift: contrary to the phase-advanced circadian clock in the liver and WAT, the relative abundance of butyric acid-producing family *Clostridiaceae_1* was significantly phase-delayed in the HF group compared with the Ctr group (50); (2) loss rhythmicity: *Veillonellaceae* and *Prevotellaceae* have been confirmed to be responsible for producing SCFAs (51, 52), and both lost rhythmicity in the HF group; (3) gain rhythmicity: the families *Erysipelotrichaceae* and *Ruminococcaceae* both gained rhythmicity in the HF group compared with the Ctr group. The study has revealed that *Erysipelotrichaceae* accelerated cholesterol accumulation by producing trimethylamine *N*-oxide, indicating its role in modulating lipid metabolism (53). Furthermore, many genera from the *Ruminococcaceae* family are SCFA producers that alter SCFA production and thereby influence systemic circulation (54). Studies have the alteration of the gut microbiota in the HF in the gestational mice (44, 45). To date, several studies have explored the diurnal rhythm of the gut microbiota in response to the HF (17, 18, 23). However, data identifying the classical markers in the circadian rhythm in the HF directly in the pregnant mice are lacking. Hence, for the first time, our study revealed that the HF distinctively altered the rhythmic pattern of the gut microbiota. Given that all of the altered families belong to the SCFA-producing bacteria, we were triggered to detect the concentration and rhythmic pattern of SCFAs.

SCFAs, produced by intestinal microbial fermentation of dietary fibers and complex carbohydrates, consist primarily of acetic acid, propionic acid, and butyric acid (55). SCFAs can be transported by the portal vein to peripheral tissues, serving as mediators of the cross talk between the gut microbiome and other organs (56). Since SCFAs are mainly absorbed by the liver (17, 34), we monitored the hepatic SCFA content. Among all the SCFAs, butyric acid exhibited a diurnal rhythm in both feeding protocols, and its rhythmic phase was significantly delayed in the HF group compared with the Ctr group. As a kind of energy source, butyric acid is considered an important regulator of gene expression and appears to play essential roles in gut health and host metabolism (57). In human and mouse B cells, butyric acid inhibited the expression of genes *Aicda* and *Prdm1* by acting as histone deacetylation inhibitor (58). Leone et al. reported that butyric acid caused alteration of the diurnal pattern of the hepatic circadian clock, mainly by impacting the expression of *Per2* and *Bmal1* in mouse hepatic organoids (17). A recent study revealed that increased butyric acid dramatically expanded the phase and amplitude of hepatic *Per2* expression, thus enhancing the expression of the downstream phospholipid metabolic genes, enabling the host to maximize caloric uptake (59). Interestingly, for the first time, our study found that the phase alteration of butyric acid was consistent with that of the butyrate-producing family *Clostridiaceae_1* in the gestational mice. The correlation analysis further confirmed the association between them by showing that butyric acid was significantly positively correlated with *Clostridiaceae_1*. These data may provide support for the hypothesis that the HF modulates the peripheral circadian rhythm by modulating gut microbiota-derived SCFAs, although assertion of a cause and effect relationship will require further investigation.

Overall, this study showed that the HF before and during pregnancy significantly induced obesity and worsen glucose tolerance, insulin sensitivity, and lipid metabolism in the gestational mice. Furthermore, we first established a gestational mice model in a circadian manner, and we found the HF resulted in a significant phase shift in hepatic and adipose circadian clock genes and downstream metabolic genes. Moreover, we performed the analysis of rhythmicity using the latest and highly sensitive tool CircaCompare. Compared with the traditional tools for rhythmicity detection, such as JTK_Cycle, Bio_Cycle, and RAIN, CircaCompare provides a means to quantify and statistically support differences between rhythms, specific to the characteristic desired (MESOR, amplitude, and phase) with high sensitivity (33). To the best of our knowledge, it is the latest tool for comparison between rhythms directly (60, 61). We took advantage of this tool to explore the circadian characteristics in the gestational mice. Importantly, our results revealed that the HF altered the diurnal rhythm of gut microbiota in a diverse manner in the gestational mice, which we classified into three categories: phase shift, loss rhythmicity, and gain rhythmicity. More fundamentally, this study reported a parallel alteration of the rhythmic phase of butyric acid- and butyrate-producing *Clostridiaceae_1*, which was further confirmed by a significant positive correlation between them. Since chronotherapeutics holds great promise for improving efficacies, these data provided support for the consideration of applying the timed treatments for managed health during gestation.

Consequently, our research emphasized the importance of the rhythmicity of gut microbiota-derived SCFAs in mediating circadian disruption in response to an HF in gestational mice, which may provide novel insights into the prevention and treatment of gestational obesity.

## Data availability statement

The data presented in this study are deposited in the NCBI repository https://www.ncbi.nlm.nih.gov/, accession number: PRJNA846143.

## Ethics statement

The animal study was reviewed and approved by the Animal Care and Ethics Committee at Peking Union Medical College Hospital (Beijing, China, XHDW-2019-012).

## Author contributions

LD was responsible for the animal experiments, data analysis, making figures, drafting, and revising the manuscript. JL helped with the study design and reviewed the manuscript. XJ helped with the data analysis. LZ, SL, QZ, and MY helped with the animal experiments. XX contributed to the whole study design, data interpretation, and reviewed the manuscript. All authors have read and approved the final version of the manuscript.

## Footnotes

1
http://qiime.org/scripts/split_libraries_fastq.html

2
http://drive5.com/uparse/

3
http://www.arb-silva.de/
