# Supplementary material for: A high-fat diet disrupts the hepatic and adipose circadian rhythms and modulates the diurnal rhythm of gut microbiota-derived short-chain fatty acids in gestational mice
Source: Front Nutr. 2022 Sep 28;9:925390. doi: 10.3389/fnut.2022.925390 (PMC9554467; doi:10.3389/fnut.2022.925390)
Supplement: Supplementary file 1 [file Table_1.DOCX]

**Supplementary Material**

**Table S1. Primers used in this study.**

| Genes | Primers |
| --- | --- |
| *Clock* | forward: AGACGGCGAGAACTTGGCATTG  reverse: TTCCTTGAGACTCACTGTGTTGATACG |
| *Bmal1* | forward: CCACCTCAGAGCCATTGATACA  reverse: GAGCAGGTTTAGTTCCACTTTGTCT |
| *Per2* | forward: GCTGCGGATGCTCGTGGAATC  reverse: GGTTGTGCTCTGCCTCTGTCATC |
| *Cry2* | forward: TGGACAAGCACTTGGAACGGAAG  reverse: GTAGAAGAGGCGGCAGGAGAGG |
| *Rev-erbα* | forward: TACATTGGCTCTAGTGGCTCC  reverse: CAGTAGGTGATGGTGGGAAGTA |
| *PPARα* | forward: TCGAGGAAGGCACTACACCT  reverse: CCAGGCTACAGTGGGACATT |
| *SREPB1* | forward: AGGTGTATTTGCTGGCTTGGT  reverse: AGAGATGACTAGGGAACTGTGTGT |
| *PGC-1α* | forward: AGCCGTGACCACTGACAACGAG  reverse: GCTGCATGGTTCTGAGTGCTAAG |
| *Glut2* | forward: TCAGAAGACAAGATCACCGGA  reverse: GCTGGTGTGACTGTAAGTGGG |
| *G6P* | forward: CCTCCTCAGCCTATGTCTGC  reverse: AACATCGGAGTGACCTTTGG |
| *ACC* | forward: GATGAACCATCTCCGTTGGC  reverse: GACCCAATTATGAATCGGGAGTG |
| *ATGL* | forward: TTCACCATCCGCTTGTTG  reverse: AGTTCCACCTGCTCAGAC |
| *PPARγ* | forward: TCGCTGATGCACTGCCTATG  reverse: GAGAGGTCCACAGAGCTGATT |
| *HSL* | forward: GCTGGAGGAGTGTTTTTTTGC  reverse: AGTTGAACCAAGCAGGTCACA |
| *PPIA* | forward: GAGCTGTTTGCAGACAAAGTTC  reverse: CCCTGGCACATGAATCCTGG |
| *β-actin* | forward: GTCCACCTTCCAGCAGATGT  reverse: GAAAGGGTGTAAAACGCAGC |
